# The Dental Protective Association

**Published:** 1890-05

**Authors:** 


### The Dental Protective Association.

In a communication recently received from Dr. Crouse, we are pleased to learn that the work of that association is going on very satisfactorily. The accumulation of evidence now on hand places the final result of its work, so far as the patents of the International Tooth Crown Company are concerned, beyond a question; that is, if the opportunity to test the character of this evidence is ever given by further prosecution, by the International Tooth Crown Company.

The mempership of the Protective Association is constantly increasing and is very large. Every member of the dental profession ought to see that it is for his interest to become a member of this protective association. In doing so, one is placed at ease in reference to future annoyance. Every member of this association will be afforded full protection, should prosecution be made. Those remaining outside, of course must submit to whatever exactions may be made upon them, or fight solitary and alone, or refrain from using any method or process upon which the International Tooth Crown Company can make a claim. We would suggest that the mempership fee ($10.) is a very small consideration for that which is secured by it, viz.: freedom from harrassing agents, exorbitant demands, and perhaps prosecution.

Every one should remember that the profession has a future in which we should all feel interested, and if organizations such as the Old Dental Rubber Company and the International Tooth Crown Company are permitted to have full sway, who can imagine what that future may be? A company would be formed in regard to every little matter upon which a patent might be obtained, and the profession would ultimately become, in a large measure, subservient to the demands of such organizations. Let the profession, once for all, promptly come up to the work and put a stop to this state of affairs. If the men who are conducting the work of this protective association are willing, and actually are devoting a large share of their time and energies to it, should not the members of the profession be willing to promptly contribute the small amount of the membership fee. For the benefit of those who may not be fully informed on this subject we will state that full information can be received by addressing Dr. J. N. Crouse, No. 2231 Prairie Avenue, Chicago.

